# Professional disrespect between doctors and nurses: implications for voicing concerns about threats to patient safety

**DOI:** 10.1108/JHOM-06-2023-0167

**Published:** 2024-08-12

**Authors:** Emmanuel Kwasi Mawuena, Russell Mannion, Nii Armah Adu-Aryee, Francis A. Adzei, Elvis K. Amoakwa, Evelyn Twumasi

**Affiliations:** Department of Organisational Behavior and HRM, Sheffield Hallam University, Sheffield, UK; Health Services Management Centre, University of Birmingham, Birmingham, UK; University of Ghana Medical School, University of Ghana, Accra, Ghana; Department of Health Services Management, University of Ghana, Accra, Ghana; UCEN Manchester, Manchester, UK; Department of Management Science, University of Education Winneba, Winneba, Ghana

**Keywords:** Disrespect, Voice and silence, Patient safety, Doctor-nurse, Surgery

## Abstract

**Purpose:**

Previous research has demonstrated that social-relational factors are instrumental to employee voice. An essential aspect of this relates to notions of respect or disrespect. Although nurses commonly report experiencing professional disrespect in their interaction with doctors, earlier studies have focused on how the professional status hierarchy and power imbalance between doctors and nurses hinder speaking up without considering the role of professional disrespect. Addressing this gap, we explore how professional disrespect in the doctor–nurse relationship in surgical teams influences the willingness of nurses to voice legitimate concerns about threats to patient safety.

**Design/methodology/approach:**

Fifty-seven semi-structured interviews with nurses drawn from a range of specialities, ranks and surgical teams in three hospitals in a West African Country. In addition, two interviews with senior representatives from the National Registered Nurses and Midwifery Association (NRNMA) of the country were undertaken and analysed thematically with the aid of NVivo.

**Findings:**

Disrespect is expressed in doctors’ condescending attitude towards nurses and under-valuing their contribution to care. This leads to safety concerns raised by nurses being ignored, downplayed or dismissed, with deleterious consequences for patient safety. Feeling disrespected further motivates nurses to consciously disguise silence amidst speech and engage in punitive silence aimed at making clinical practice difficult for doctors.

**Originality/value:**

We draw attention to the detrimental effect of professional disrespect on patient safety in surgical environments. We contribute to employee voice and silence by showing how professional disrespect affects voice independently of hierarchy and conceptualise the notion of punitive silence.

## Introduction

Employee voice is a topic that has attracted much attention across a range of management disciplines (Mawuena and Wilkinson, 2024; Wilkinson *et al.*, 2020). In this study, we define employee voice as the discretionary expression of ideas, inputs or suggestions to persons who might be able to take appropriate action, leading to improvements in work outcomes (Milliken *et al*., 2003; Morrison, 2023; Van Dyne *et al*., 2003; Morrison *et al*., 2015). In contrast, employee silence occurs when information or ideas are consciously withheld, potentially depriving organisations of opportunities to bring about improvement or change (Morrison and Milliken, 2000). Voice and silence are not limited to the presence or absence of speech as employees may not speak up because they lack sufficient knowledge to ascertain what needs correction. Employees may also express their voice while simultaneously withholding important information required to improve work outcomes (Morrison, 2014; Van Dyne *et al*., 2003; Pinder and Harlos, 2001). Voice and silence behaviour has therefore been described as a multifaceted phenomenon representing two sides of the same coin.

Research across a range of industries and sectors has demonstrated that power differences within a hierarchy present numerous barriers to voice, including a predilection for superiors to ignore, downplay or not act appropriately on employee voice (Pfrombeck *et al*., 2022; Van Dyne *et al*., 2003; Morrison *et al*., 2015). Within healthcare organisations where professionals are organised into professional hierarchies, medical specialities are dominant. For example, nurses are expected to work under the direct supervision of doctors (Freidson, 1988; Abbott, 2014). There is now a large body of evidence that nurses’ voice on patient safety lacks efficacy compared to that of doctors (Churchman and Doherty, 2010; Morrow *et al*., 2016; Schwappach and Gehring, 2014). Although voice has been widely studied, the social-relational antecedents of voice remain under-researched (Ng *et al*., 2021), particularly, in the doctor–nurse relationship. Here, we focus on an important aspect of social relations between doctors and nurses – professional disrespect.

## Social-relational antecedents to voice and silence

Research has demonstrated that employees tend to express voice when they enjoy good relationships (Milliken *et al*., 2003; Burris *et al*., 2008; Botero and Van Dyne, 2009). While the support of colleagues and supervisors appears to create a strong sense of obligation that enhances promotive voice (Xie *et al*., 2015; Burris *et al*., 2008), abusive and disrespectful supervisory relationships undermine motivation for voice (Farh and Chen, 2014). These align with the notion of reciprocal expectations in social relationships (Blau, 1964; Cropanzano and Mitchell, 2005) where employees are inclined to utilise voice when they feel satisfied in their employment and valued by superiors. It is also consistent with the idea of voice signifying respect and dignity of employees as part of organisational fairness (Budd, 2019). This foregrounds the importance of social-relational antecedents to voice, as voice occurs relationally between receivers and voicers (Bashshur and Oc, 2015). However, more sustained research is needed to better understand the social-relational contexts which motivate employees to volunteer or withhold voice (Ng *et al*., 2021).

A literature review by Chamberlin *et al*. (2017) identified 8 social-relational factors out of 32 main predictors of voice and silence. Of these, 2 related to peers’ social support and group identity, and 6 related to styles of leadership (leader–member exchange, transformational leadership, leader openness, ethical leadership, abusive supervision and trust in leader). Although employees value respect, making it an essential variable in explaining workplace behaviours (Rogers *et al*., 2017; Van Quaquebeke *et al*., 2009) such as voice, interprofessional respect has received scant attention in the literature. In a study exploring employee–coworker and subordinate–supervisor relations, Ng *et al*. (2021) found that when employees feel respected and valued by coworkers and supervisors this engenders a positive psychological mood which motivates voice and enhances listening to voice. While a recent review by Morrison attests to considerable research progress in understanding the relational antecedents to voice (Morrison, 2023), there remain limitations in current studies.

First, research has demonstrated that positive social-relational experiences promote voice (Ng *et al*., 2021; Xie *et al*., 2015). The inference is that employees who do not experience or perceive positive social-relational factors will withhold voice from a lack of engagement (Van Dyne *et al*., 2003). Studies have therefore focused on the social-relational effects from voicers’ perspective without addressing how this might influence attitude or receptivity to voice from targets’ perspectives. For instance, team members who are disrespected and whose knowledge or contributions are not valued are less likely to be taken seriously when they do speak up. Second, drawing on Social Exchange Theory (Blau, 1964), we argue that social-relational experiences such as disrespect might be reciprocated negatively and manifest in more nuanced forms of voice behaviour beyond mere silence. Third, research has focused on social-relational factors to voice in employer–employee, superior–subordinate and to some extent peer relationships (Xie *et al*., 2015; Ng *et al*., 2021; Burris *et al*., 2008) but has largely ignored these factors in team-oriented settings that are ubiquitous in healthcare services.

## Social-relational, disrespect and voice and silence in healthcare

Medical settings are anchored in complex embodiments of historical and social-relational working conditions, which can facilitate or inhibit speaking-up behaviours (Szymczak, 2016). The Doctor–Nurse Game has been used as a simple heuristic to describe how doctors and nurses engage in stereotypical patterns of social interaction (Stein, 1967). Here, nurses offer subtle non-verbal and verbal advice while giving the impression of deferring passively to doctors’ authority to maintain doctors’ esteem and avoid challenging their authority. While it has been claimed that decades of nursing education and interprofessional collaborations have made the doctor–nurse relationship less hierarchical and more collegial (Pritchard, 2017; House and Havens, 2017; Ernst and Tatli, 2022), there is convincing evidence that in many healthcare settings, little has changed in this regard (Reed, 2016; Center for Health Ethics, 2023).

According to Espin *et al*. (2006), individual-cognitive and organisational determinants of voice do not fully account for the social allegiances, friendships and conflicts that shape the delivery of care. For instance, mutual respect is widely regarded as essential to improving relational coordination and communication among multidisciplinary surgical teams (Tørring *et al*., 2019) while disrespectful behaviours pose a threat to patient safety (Leape *et al*., 2012). Stievano *et al*. (2018) found that nurses valued respect in professional relationships and being recognised and valued for their contributions. Indeed, nurses consider being respected as the most important factor in their contribution to surgical teams (Kaldheim and Slettebø, 2016), and disrespect for nurses is associated with poor patient outcomes (Jackson *et al*., 2013; Laschinger, 2014). However, there is widespread evidence of doctors’ disrespect for nurses, including unprofessional behaviours such as bullying, harassment, microaggressions and rudeness (Stievano *et al*., 2018; Sirota, 2008; Maben *et al*., 2023).

Nurses’ experience of disrespect is further linked to high levels of esteem and privileges accorded to doctors in the healthcare system and wider society. Most medical institutions are organised on the assumption that doctors are the primary decision-makers with ultimate responsibility for patients (e.g. Lancaster *et al*., 2015; Nugus *et al*., 2010). This is usually justified on the basis of the duration of doctors’ medical training (e.g. Hall, 2005; Baker *et al*., 2011). Therefore, nurses’ knowledge, experience and competencies are often downplayed and perceived as inferior to those of doctors (Aveling *et al*., 2015; Abbott, 2014; Schwappach and Gehring, 2014). Although the social status and prestige traditionally enjoyed by doctors are said to be diminishing, especially in Western countries (McKinlay and Marceau, 2002), doctors continue to be treated more favourably and occupy positions of enhanced prestige and respect compared to nurses (Abbott, 2014; Center for Health Ethics, 2023).

However, research has focused on how the traditional status hierarchy between doctors and nurses undermines nursing voice while paying little attention to the role of professional disrespect. This focus might be flawed by over-attributing silence to hierarchy. From the perspective of Social Exchange Theory (Blau, 1964), disrespect might affect voice independently or reinforce hierarchical barriers to voice. For instance, disrespect for nurses, including under-valuing their contribution to care means their voice is likely to go unheeded. Again, in relation to the multifaceted nature of voice and silence (Morrison, 2014; Van Dyne *et al*., 2003; Pinder and Harlos, 2001), disrespect might engender a more subtle or punitive silence, such as actively withholding important information. Although the Doctor–Nurse Game typically describes nurses’ attempts to indirectly correct doctors or provide input in ways which do not usurp the authority of doctors (Stein, 1967), feeling disrespected might motivate nurses to abdicate responsibility to doctors through talking around problems without identifying causes and solutions to them. We, therefore, address this gap in the literature by exploring how professional disrespect in the doctor–nurse relationship within surgical teams influences nurses’ voice behaviour with regard to threats to patient safety.

We contribute knowledge on the social-relational antecedents to voice (Ng *et al*., 2021; Morrison, 2023; Xie *et al*., 2015) through the lenses of professional disrespect and voice behaviour regarding threats to patient safety. We further provide theoretical insight into the multifaceted nature of voice and silence (Van Dyne *et al*., 2003; Morrison, 2014; Pinder and Harlos, 2001) and contribute nuanced insights into the doctor–nurse relationship (Pritchard, 2017; House and Havens, 2017; Morrow *et al*., 2016; Stein, 1967; Ernst and Tatli, 2022).

### Social Exchange Theory

Social Exchange Theory is a sociological and psychological theory that seeks to understand and explain social and relational behaviour between two interacting parties. The theory posits that relationships evolve and develop over time depending on how each party abides by explicit or implicit rules (Blau, 1964). Implicit in exchange rules that govern behaviour is the extent to which both parties reciprocate positive or negative behaviours such as displaying mutual respect, affection or disrespect (Blau, 1964; Cropanzano and Mitchell, 2005). For example, a good deed may attract favourable treatment from the other party, while an offensive act may attract negative treatment (Cropanzano and Mitchell, 2005). Social Exchange Theory suggests that the use of voice is largely others-oriented (Ng and Feldman, 2012). Employees who are satisfied with their jobs and feel valued reciprocate by voicing constructive ideas and suggestions to improve team and organisational functioning and outcomes (Ng and Feldman, 2012; Burris *et al*., 2008; Van Dyne and LePine, 1998). These studies indicate that an individual’s willingness to exercise voice or remain silent is dependent on the quality of social exchange between parties; for example, whether relationships are characterised by mutual trust or distrust, respect or disrespect and satisfaction or dissatisfaction. The Social Exchange Theory perspective on voice has been criticised for focusing on others-oriented voice while voice can be self-interested (Ng and Feldman, 2012). However, as healthcare professionals largely speak up in the interest of patients rather than themselves (Okuyama *et al*., 2014), we consider Social Exchange Theory as suitable for exploring voice behaviour in the doctor–nurse relationships.

## Methods

### Study design and setting

From a social constructivist perspective, we used a qualitative study design, comprising semi-structured interviews to explore social-relational issues (Saldana, 2011) pertinent to voice and silence in surgical teams in three hospitals in a West African Country. To provide context, 2 semi-structured interviews were also undertaken with representatives from the National Registered Nurses and Midwifery Association (NRNMA) of the country. The participating hospitals comprise two Teaching Hospitals (Hospital HK and Hospital HM) and a private hospital (Hospital HD). Hospital HK has about 1,000 beds and 7 specialised surgical units. Hospital HM has about 300 beds and 5 specialised surgical units. Hospital HD is a privately owned facility, which undertakes general surgeries and has about 30 beds.

### Sampling and data collection

The data is a part of a larger project. Purposive sampling was used to ensure the inclusion of the perspectives and experiences of nurses covering different specialities and ranks. Although we aimed to include a proportional sample of junior and senior ranks, based on emerging responses, we decided to include more senior ranks to ensure that responses were not only a reflection of junior nurses’ experience. After 57 interviews no new insights emerged and data saturation was achieved. A detailed breakdown of the sample is presented in Table 1 below.

Following interviews with members of surgical teams in the three hospitals, two interviews were undertaken with senior executives from the NRNMA to corroborate the emerging issues. Out of the total 59 participants, 13 (22%) were males and 46 (78%) females. Twenty-two (37%) were junior ranks and 37 (63%) were senior ranks. Participants’ years of work experience ranged from 1 to 35 years with an average of 11 years.

Face-to-face interviews were conducted. These lasted between 40 and 70 min. All interviews were conducted in English and participants’ consent was obtained for interviews and recordings. Interview questions were guided by voice and silence questions as set out in Schwappach and Gehring (2014). The interviews began by confirming background information, such as participants’ rank and roles. Specific questions asked included: how comfortable participants were in raising patient safety concerns; examples of where participants had raised safety concerns; and instances of when they felt inhibited in voicing safety concerns. The interviews also probed the circumstances surrounding voice and silence, including who was involved, what was the outcome or response and whether the situation was resolved. From these, the social-relational factors relating to professional disrespect emerged and was deeply probed. The follow-up interviews with the representatives of NRNMA explored broader questions on the doctor–nurse relationship and verified the key issues that emerged from the main interviews.

### Analysis

Our analysis is based on the perceptions and subjective experiences of individuals. Interviews were fully transcribed and read to enhance familiarity and then uploaded into NVivo. The interview transcripts were open-coded by breaking down responses into recurrent patterns, issues and ideas in the words of participants. Next, initial codes were examined in detail to surface common underlining patterns through an inductive and iterative approach involving a series of back-and-forth movements between the data and literature to revise codes into broader patterns (e.g. Ritchie *et al*., 2014) leading to the second and the third order codes (the themes). Initial coding and analysis were undertaken by the first author. The second author independently analysed half of the transcripts to verify the emerging codes and worked closely with the first author to refine the themes. The other co-authors provided feedback, which was incorporated into the final themes namely – A culture of venerated doctors and undervalued nurses; Doctors’ Disrespect for Nurses; Nurses Silenced – Alternative Strategies to Safety and Distress; Nurses’ Punitive Silence in Response to Disrespect. This study was approved by the ethics committees of the participating hospitals. Quotes are used to illustrate each theme and labelled with the professional group of the interviewee. The quotes have been slightly edited for language and flow.

## Findings

### A culture of venerated doctors and undervalued nurses

Consistent with doctors’ high levels of social and professional esteem (Abbott, 2014; Center for Health Ethics, 2023), nurses described wide disparities in the privileges and authority accorded to doctors compared to themselves within and outside healthcare settings. Nurses frequently reported that doctors are cast in the image of all-knowing and virtuous ‘demi-gods’ who are treated with reverence and respect in social and professional spaces. This contrasted with nurses who felt frequently disrespected both at work and by the public. Indeed, nurses described frequent instances of derogatory behaviour and attitudes by patients, including verbal abuse and physical assaults.

You see in country X there is this general perception among the public that doctors do no wrong and nurses are the bad ones. So, if something bad happens it is the nurses (Senior-Theatre-Nurse-2-HM)

We have had abuses to the extent of patients slapping nurses (Ward-Nurse-2-HD)

Nurses described how the privileged treatment of doctors was manifest at all levels in healthcare. A nurse described how a cleaner who was well known for reprimanding staff and patients from walking across floors while mopping but looks on without saying a word when doctors walk across. While nurses reported that they did not expect to have the same privileges as doctors, many thought that the level of disparity was unacceptable and undervalued their contribution.

They motivate doctors and leave others. It leaves people with some sort of anger. We know you are doctors – we cannot rub shoulders with you but why do they motivate them and leave others out completely? (Senior Nurse-Anaesthetist-1-HK)

Moreover, nurses described a widespread reverence for doctors’ knowledge and decision-making authority. It was reported that doctors summarily dismissed nurses’ expertise by frequently referring to the many years of medical training and education they had undertaken. Nurses described the high professional and social status of the medical profession as being institutionally entrenched and reinforced by unwritten rules that create an aura of power and respect for doctors which was difficult to challenge.

If you are not a doctor, then forget it. You need to humble yourself because the doctors see themselves as superior and expect certain things from you – the way you talk with them in the theatre. In a way, they want to be worshipped (Senior Nurse-Anaesthetists-1-HK)

I may not be able to pinpoint where exactly it is coming from, but it is just an unwritten something – the doctors have it at the back of their minds they are the bosses, and the nurses have their own place (Senior-Nurse-Anaesthetist-1-HM)

### Doctors’ disrespect for nurses

Nurses frequently use the word ‘respect’ in describing the importance of being treated with dignity, being valued as professionals and for their contribution to patient care.

To help improve patient safety there should be respect and recognition of everybody (Senior-Nurse-Anaesthetist-2-HM)

The whole concept of teamwork is key, respect for views closes all the communication gaps (NRNMA-1)

Although some nurses reported that they felt respected by some doctors, especially those with who are friendly or have a ‘nice’ personality, they shared a general pattern of being disrespected and undervalued.

A few doctors are very approachable. They listen, they treat you with respect and dignity (Senior-Theatre-Nurse-5-HM)

Some listen to you and answer your questions, but others will give you cheeky answers (Junior-Ward-Nurse-1-HD)

It was reported that those nurses recognised as being particularly knowledgeable and those with strong and assertive personalities tend to garner respect from doctors. It was noted that junior doctors who often depended on nurses for guidance and showed respect to them, often became more disrespectful to nurses once they had fully qualified as doctors.

Most house officers are down to earth because they don’t know much. So, they give that respect to the input from nurses, they seek advice and learn along the way till they themselves graduate to become medical doctors. There are some who also get to that stage, and they think they don’t need anybody’s guidance or advice anymore. They feel they know (NRNMA-2)

Nurses shared their experiences of frequent disrespect, such as being ‘yelled at’ and told to ‘shut up’ when they raised concerns about patient safety:

There was a recent case where a nurse was told to shut up by a surgeon for suggesting something. And the nurse also told the surgeon to shut up. But the nurse was reported and was given a query letter to respond to, for also telling the surgeon to shut up (Senior-Theatre-Nurse-2-HK)

Here on this ward, some doctors even abuse nurses. Yes, they do! (Senior-Ward-Nurse-3-HM/Manager)

Nurses reported they are often talked down to and treated as inferior team members who know ‘little or nothing’. As a result, while nurses possess in-depth knowledge about patients from their close contact with them, they often do not share this. Some nurses likened their experience of Ward Rounds to ‘zombies’ who follow doctors but are rarely involved in discussions or listened to when they express an informed professional opinion.

Some of the doctors feel that nurses are not learned as they are. Some ask, did you go to medical school? Did you study anatomy? (Junior-Theatre-Nurse-1-HD)

A theatre nurse recounted how his offer to assist in suturing following a long surgical procedure in order to save time was dismissed. The nurse reported that although he is experienced in suturing, the surgeon rejected the offer of help stating that ‘he does not even allow house officers to suture, much less ‘a nurse’. Similarly, a nurse anaesthetist said he was ‘told off,’ when he offered to help a visiting physician anaesthetist who was struggling to set an IV line for a baby.

As soon as I touched the patient’s hand, the visiting anesthetist told me that the patient is not a ‘punching bar’ for us to be poking with needles (Senior-Nurse-Anaesthetist-3-HM)

According to the nurse, he stepped aside and observed the doctor struggle until his assistance was requested. As reported, he obliged and was successful at the first attempt, much to the doctor’s embarrassment.

### Nurses silenced: alternative strategies to safety and distress

We found that doctors’ authority, status and general disrespect for nurses undermined effective teamwork and suppressed voice. Reflecting the socialisation of nurses into doctors’ authority (Roberts, 2006), some nurses showed deference towards doctors and their knowledge, by remaining silent. It was reported that even nursing managers are often cowed by doctors’ authority into silence, with only a few effectively challenging the authority of doctors.

Even matrons who are the head of the nurses sometimes are afraid to talk to doctors. They are afraid to voice out because they see them as smaller gods. It is a problem; it is a problem here in Africa (Senior-Nurse-Anaesthetist-1-HK)

However, we found that the nursing manager in the private hospital had more authority over the day-to-day management of the hospital, including control over doctors’ behaviour. Because most doctors worked at the hospital on a part time basis, this enhanced the nursing manager’s authority over doctors relative to nursing managers in public hospitals. Despite this, the NRNMA expressed a general concern about nursing managers losing their authority in relation to doctors.

Nurse managers are losing their voice when it comes to issues with doctors. It seems as if doctors own the ward and they come in and do whatever they want and go without recourse to the nurse managers (NRNMA-1)

We found that although considerable nurses were confident about their knowledge and voiced safety concerns in core nursing domains, they remained silent on those perceived as core doctors’ roles as they do not feel valued, or obliged to speak up on these or think concerns will be listened to.

In the theatre I can’t really talk about that one. But once it is coming to my side [recovery] I will step in and talk (Senior-Recovery-Ward-Nurse-5-HK)

When surgeons decide who is going to the theatre, who are you to ask why they are taking that patient to theatre and probably that the patient doesn’t require surgery? (Ward-Nurse-4-HK)

While nurses exercise voice, especially in areas relating to their speciality, this often went unheeded by doctors. Nurses reported that doctors do not feel obliged to consider their contributions and tend to downplay or dismiss them. It was reported that many doctors perceive nurses as mere ‘helpers’ or ‘handmaidens’ and view themselves in a position of overall control. Nurses highlighted how some doctors, especially senior doctors often failed to adhere to patient safety protocols, including rules about handwashing and following safety checklists.

Some surgeons will come and want to be quick. They do not take time to do surgical handwashing. When you prompt them, they will literally say we are in a hurry, and they are gone (Junior-Theatre-Nurse-10-HK)

It was claimed that failure to act upon nurses’ concerns had resulted in irrecoverable harm to patients and some deaths. A nurse recounted that based on the conditions of a patient with chest pains, she recommended giving Pethidine through Intramuscular Injection (IM) instead of Intravenous (IV) prescribed by a surgeon. However, according to the nurse, the recommendation was ignored leading to the death of the patient. In another case, a nursing manager is reported to have challenged a doctor’s drug prescription and the doctor responded angrily that it is not the nurse’s duty to tell him what to do. The nurse, therefore, declined to administer the drug and left the doctor to do it. According to the reporter, the patient died from an adverse reaction to the drug.

The doctor got angry and told the nurse in-charge that it is not her duty to tell him [doctor] what to do. He administered the drug himself and then the patient passed away (Senior-Ward-Nurse-2-HD)

In addition, reflecting the entrenched doctors’ authority over nursing practice (Reed, 2016), nurses reported how doctors pressure them into having to compromise safety standards in primary nursing roles. Nurses reported that surgeons often coerce them into unsafe procedures, including preparing non-emergency patients for surgery in haste without undertaking appropriate tests.

I don’t know how to put it. But a doctor is a doctor. It’s like I am the doctor and the head of this team, what I want is what goes. And you are the nurse. So, I am saying do this and do that. It may be outside your domain or in your domain, but I am the doctor I am ordering you to do it! (Senior-Nurse-Anaesthetist-1-HM)

You know surgery is something very critical – you don’t just wake up and go for surgery. But the next day you will be there, and a patient will come from nowhere with a folder and a surgeon tells you to prepare this patient for surgery. But you are to do it! (Senior-Ward-Nurse-1-HM)

In the two Teaching Hospitals, nurse anaesthetists are headed by a physician anaesthetist in the anaesthesia department. Nurse anaesthetists, therefore, do not have direct decision-making authority with doctors. Meanwhile, consistent with how surgeons and physician anaesthetists contest and defend care in their own speciality (Mawuena and Wilkinson, 2024), it was reported that nurse anaesthetists sometimes obtain the support of physician anaesthetists which buffers the controlling attitude of surgeons on anaesthesia issues. However, it was also reported that surgeons and physician anaesthetists sometimes collude in coercing nurse anaesthetists into compromising standards of patient safety. Nurse anaesthetists reported that when they refer patient safety concerns to physician anaesthetists, surgeons sometimes talk with physician anaesthetists, who then force them into undertaking what in their opinion were unsafe procedures. We note that this might sometimes be motivated by how pervasive resource constraints in Low-Middle-Income healthcare contexts incline leaders to ignore optimal standards of care (Mawuena and Mannion, 2022). However, such attitudes were thought to disrespect nurses and their knowledge.

I might have a case on my list and the patient’s condition is not ideal for surgery. So, I will call my superior and inform him why I don’t want to do the anaesthesia. But surgeons will call them and say – Charlie, I have this case I want to do, and the nurse anaesthetist is proving stubborn. Then they will call. And all he says is direction from above – your superior says do the case! (Senior-Nurse-Anaesthetist-3-HK)

Sometimes there are some cases you feel you don’t have to do because the patient has safety issues but because the surgeon is a consultant or senior specialist, he will go behind you and call your bosses elsewhere and tell them something and your boss will call you okay, do it in my name …. you are forced to do it (Senior-Nurse-Anaesthetist-7-HK)

## Alternative strategies to safety and distress

We found that nurses adopted a range of alternative strategies to mitigate risk to patients. Nurses reported using patient information in managing safety on their own in isolation from doctors. At other times, it was reported that nurses pass safety information to senior nurses or doctors they relate well with and trust in attempts to address safety concerns.

So, they often prefer to pass concerns through people and sometimes me as in-charge (nursing manager) to doctors because it is easier for me to talk to them – (Surgical-Ward-Nurse Matron-1-HK)

We sometimes work in our own corner, do what we can do to help patients, it is not the best but better than nothing – (Recovery/Ward-Nurse-6-HM)

Some nurses reported speaking up respectfully to doctors using a ‘friendly’ tone of voice, being indirect in their suggestions, and refraining from shouting or acting in any way that may be perceived as usurping doctors’ authority. Reflecting the Doctor–Nurse Game (Stein, 1967), it was reported that some nurses disguised suggestions in the form of questions or offered their advice using words such as– “the other time this is how this or that doctor did it”.

The best you can do is to tell them by effective communication in quote – by putting it in such a way that it doesn’t seem you are denigrating authority. So, you put it in a milder way even though it could have been put right as it is when you are in a different setting (Senior-Nurse-Anaesthetist-3-HM)

It is about the ability to say it without embarrassing doctors. This often gives way to some hearing of your view – (Peri-Operative-Nurse/Matron-1-HK)

However, participants did not use official channels to air their concerns. Consistent with the lack of formal organisational support for voice in developing healthcare contexts (Mawuena, 2020), nurses noted there are no clear policies or official reporting systems to air concerns. According to them, although errors are sometimes reviewed, powerful individuals and groups, such as doctors, are rarely implicated when patient harm arises. As nurses feel underprivileged in relation to powerful and revered doctors, they expressed little trust in management to respond to concerns and feared personal negative repercussions for raising concerns.

We don’t know of any organisational support for voice. Because even if there is a problem and it goes to the HOD, the HOD will defend the doctors (Senior-Nurse-Anaesthetist-7-HK)

So, I don’t know if there is any policy on that but even if there is, I don’t think anybody will count on that. I won’t take that seriously (Senior-Nurse-Anaesthetist-2-HM)

### Nurses’ punitive silence in response to disrespect

Consistent with Social Exchange Theory (Blau, 1964), nurses reciprocated doctors’ perceived disrespect by adopting an uncooperative attitude and remaining silent. It was reported that some nurses refused to work with certain doctors who were perceived as extremely disrespectful. This had the effect of ostracising specific doctors, who it was reported, then struggled to get nurses to work in their teams. We heard reports of some nurses ‘storming out’ of theatres during procedures when they felt disrespected or not listened to by doctors.

Someone can say I am not comfortable working with this surgeon because of that power driven thing and disrespect (Senior-Nurse-Anaesthetist-3-HK)

There are situations where people protest by scrubbing out of the theatre (Junior-Theatre-Nurse-3-HM)

Nurses reported various forms of attenuated voice behaviour in response to feeling disrespected by doctors. As many doctors give nurses instructions without listening or valuing their inputs, nurses reported adopting a passive attitude to care by simply following instructions and allowing doctors to take full responsibility (and the blame) when problems emerge. Silence was, therefore, common when safety concerns are perceived as doctors’ core role and where nurses are unlikely to be held responsible for any harm. Nurses also reported actively withholding information or talking around problems with doctors, without pointing to a solution, but instead, leaving it to doctors to work this out. Reflecting the multifaceted nature of voice and silence (Van Dyne *et al*., 2003; Pinder and Harlos, 2001), nurses may simultaneously be speaking up while withholding potentially useful information.

You know for doctors; they always want to be doctors. There is something that needs to be done and they think their way is right. You [the nurse] are not a doctor, they think they are the doctors, so you tell them that this is the problem, but you don’t give them the solution (Junior-Recovery-Ward-Nurse-8-HK)

If there is an issue such as patient complications, I will not go and explain it exactly how I really think. I may only say it in bullet points and ask – what should we do? (Senior-Nurse-Anaesthetist-1-HM).

While this mirrors the Doctor–Nurse Game (Stein, 1967), this manner of voice is motivated by feeling disrespected, thus motivating a punitive action rather than a polite approach to assisting doctors in solving problems. We further found that nurses engaged in a more direct punitive silence to make life difficult for doctors. While nurses reported expressing voice to respectful doctors, they elected for silence towards those perceived as disrespectful. There were reports of nurses leaving doctors to struggle during critical care scenarios or refusing to contribute useful ideas and solutions to improve patient care.

Their posture on the ward, they claim they are the champions and more learned than you. So, even though you have seen it [safety concerns] you leave it for them to see it! (Junior-Ward-Nurse-2-HM)

If you are a doctor, you come and feel you know it all. Once you declare that without nurses you can take care of your patients, the nurse will allow you to fail. But nurses take patients of respectful doctors seriously (Junior-Ward-Nurse-3-HK)

It was reported that some highly experienced nurse anaesthetists left less experienced physician anaesthetists and even sometimes more experienced ones who were perceived as disrespectful, to struggle in clinical situations. A nurse described watching three senior doctors fail to deliver a spinal anaesthesia several times due to a poor positioning of the patient, without speaking up due to the doctor’s perceived superior posturing. According to the nurse, he was only obliged to ‘save the situation’ when the doctors requested his assistance.

So, when I came in, I changed the position – because I knew the position of the patient wasn’t good. and I just tried once, and I had it. I was expecting the first doctor to ask me how come you got it easily and I will tell him anaesthesia is by experience. But they didn’t ask me (Senior-Nurse-Anaesthetist-6-HK)

We, therefore, conceptualise punitive silence in the context of professional disrespect.

## Discussion and theoretical implications

Although research has highlighted the social-relational factors that shape voice and silence (Xie *et al*., 2015; Ng *et al*., 2021; Morrison, 2023), this presents only a limited conceptualisation of employee voice behaviour. In this study, we used Social Exchange Theory (Blau, 1964) as a framework to explore employee voice in the context of professional disrespect within the doctor–nurse relationship. We found that nurses frequently experience disrespect by doctors who do not value nurses’ knowledge and contributions to care. This inclined doctors to undervalue nurses’ voice which further motivates nurses to remain silent and, in some situations, expose patients to avoidable harm. Although we found evidence of the subservient role of nurses, including the traditional deference towards doctors (Stein, 1967; Roberts, 2006) leading to silence, nurses did exercise voice, especially related to core nursing roles, but their voice is often ignored or dismissed. There were reports of negative consequences including patient deaths arising from doctors ignoring the concerns of nurses. Our findings suggest that while nurses’ knowledge base may have increased, the claim of more collegial relationships (House and Havens, 2017; Ernst and Tatli, 2022) may be premature. As doctors’ knowledge status is often justified by their length of clinical training (Hall, 2005; Baker *et al*., 2011), this may be heightened in many African countries where it takes between 14 and 16 years of medical training to specialise in surgery. However, considering that doctors’ control over the work of nurses is an international phenomenon (Malloy *et al*., 2009; Center for Health Ethics, 2023; Stievano *et al*., 2018), these findings in a high-power distance national cultural context (Hofstede *et al*., 2010) are not particularly surprising. However, the situation may be difficult to address as speaking up and challenging authority is more difficult within such national cultures.

A novel and striking finding is how doctors’ disrespect motivates subtle voice behaviour among nurses. Respect is recognised as instrumental to prosocial behaviour, such as voice (Rogers *et al*., 2017; Van Quaquebeke *et al*., 2009; Ng *et al*., 2021), and disrespect is a commonly reported experience of nurses in their professional relationship with doctors (e.g. Malloy *et al*., 2009; Aveling *et al*., 2015; Sirota, 2008; Stievano *et al*., 2018; Jackson *et al*., 2013). Through the lens of Social Exchange Theory (Blau, 1964), we found that disrespect undermined nurses’ self-worth, led to apathy and motivated various forms of voice behaviour. Feeling disrespected motivated nurses to consciously talk around problems while actively withholding information that could be useful in solving problems. This provides theoretical insight into the multifaceted nature of voice and silence (Van Dyne *et al*., 2003; Morrison, 2014; Pinder and Harlos, 2001) in that feeling disrespected motivates the disguising or masquerading of silence within speech. While this manner of speech is considered to be part of the classical Doctor–Nurse Game, where nurses attempt to correct doctors in a polite manner without usurping their authority (Stein, 1967), we demonstrate how this is a subtle form of silence. Moreover, disrespect, including not valuing the contributions of nurses, motivated nurses to actively engage in punitive silence by purposefully withholding information which would have been useful for patient safety and allowing doctors to struggle in critical clinical situations. Although silence is also motivated by doctors ignoring the voice of nurses, some are purposely targeted at punishing doctors, although it is patients who may ultimately suffer the negative consequences. We, therefore, conceptualise punitive silence as a type of silence in the context of professional disrespect.

While extant research overwhelmingly focuses on hierarchical hindrances to voice (e.g., Churchman and Doherty, 2010; Schwappach and Gehring, 2014), we highlight a more subtle role of professional disrespect. The crucial role of interpersonal relationships in collectivist African societies (Hofstede *et al*., 2010) may reinforce the negative effects of professional disrespect. Our study addresses the call for research on social-relational antecedents to voice (Ng *et al*., 2021), and its effects on patient safety practices in medical settings (Szymczak, 2016; Espin *et al*., 2006). We highlight how system silencing (Donaghey *et al*., 2011) might be reinforced by social and professional systems in perpetuating silence.

### Practical implications

This study brings to the fore the significance of professional respect for teamwork and voice for the safety of patients. Although little may change in the traditional medical hierarchy, socialising health professionals into valuing the contribution of all team members irrespective of speciality may help to enhance the professional dignity and voice of nurses. Moreover, as disrespect for nurses is rooted in social and healthcare inequalities, health stakeholders, including governments, should do more to mitigate these to improve nurses’ sense of self-worth and belonging. Change in social and professional attitudes through enforceable policies against abusive behaviours will protect and promote respect for nurses and enhance their voice for patient safety. Similarly, as developing healthcare institutions often lack policies and support for voice (Mawuena, 2020), designing these to empower nurses, especially in multidisciplinary teams is crucial. For instance, a simple reporting procedure about ignored safety concerns could be used to trace and address avoidable patient harm. As collectivist African culture (Hofstede *et al*., 2010) can hinder its use, this could be best managed through anonymous online reporting. Finally, we call for medicolegal practices in developing healthcare contexts that promote medical accountability for patient care and outcomes.

### Strength, limitations and future research

As our sample is drawn from three hospitals in a high-power distance country (Hofstede *et al*., 2010), findings may not necessarily apply in low-power distance contexts. Moreover, while voice is influenced by a wide range of factors, we focused on a widely shared subject by nurses. Our study indicates that hospital contexts can empower and disempower nurses in relation to doctors and that nurses’ personalities and depth of knowledge can influence the amount of respect they receive from doctors with regard to voice. Further studies may explore how these factors enable or hinder nurses’ voice. There is also a need to understand professional disrespect, patient care and voice behaviour in other healthcare settings, especially in Western and multi-ethnic healthcare contexts.

## Conclusion

In this study, we draw attention to the negative effect of professional disrespect on effective teamwork, voice and patient safety. It points to the need for working cultures which respect all health professionals and value their contribution to patient care. This, we believe, will have a positive impact on the willingness of staff lower down the professional hierarchy to raise legitimate concerns and motivate those in positions of power to listen to and act on genuine concerns (Mannion and Davies, 2015).

## Figures and Tables

**Table 1:** Interview participants

Nursing groups	Hospital (HK)	Hospital (HM)	Hospital (HD)	Total in hospitals	Representative of the national nurses and midwifery association	Grand totals
Theatre Nurses	12	6	3	21	2	
Nurse Anaesthetists	10	3	1	14		
Recovery and Ward Nurses	13	7	2	22		
Totals	35	16	6	57		59

**Source(s):** Created by the authors
